# Nordic population-based study on internet use and perceived meaningfulness in later life: How they are linked and why it matters

**DOI:** 10.1177/1403494820987459

**Published:** 2021-02-15

**Authors:** Emilia W.E. Viklund, Ingeborg Nilsson, Anna K. Forsman

**Affiliations:** 1 Faculty of Education and Welfare Studies, Health Sciences, Åbo Akademi University, Vaasa, Finland; 2 Department of Community Medicine and Rehabilitation, Occupational Therapy, Umeå University, Umeå, Sweden

**Keywords:** Older adults, internet, gerontechnology, meaningful life, meaningful activity, Finland, Sweden, surveys and questionnaires

## Abstract

**Aim:**

The aim was to explore the association between internet use, the use of specific internet-based activities and perceiving life as meaningful, among older adults in two regions in Finland and Sweden.

**Methods:**

The data was collected through a population-based survey (*N* = 9386) as part of the GERDA project conducted in 2016. In order to analyse the associations between perceiving life as meaningful and internet use and related activities, odds ratios with a 95% confidence interval were calculated using binary logistic regression analysis, where socio-demographic factors and health status were controlled for.

**Results:**

Statistically significant associations were found between perceiving life as meaningful and internet use in later life. When looking further at the specific internet-based activities under study, activities related to leisure and entertainment showed a statistically significant connection to perceived meaningfulness in later life, after controlling for socio-demographic factors and health status.

**Conclusions:**

The results indicated that there was a statistically significant positive association between internet use and perceiving life as meaningful in later life. Online activities related to leisure and entertainment seem to be especially associated with perceived meaningfulness. Although causal direction could not be determined, the results suggest that internet use may support the experience of wellbeing in everyday life among older persons, through the unlimited access to interest-driven activities that it provides.

## Introduction

Meaning in life is often understood as having clear life missions or a sense of higher purpose [1]. However, a meaningful life does not necessarily have to entail higher causes or life missions. Life can also be perceived as meaningful if it contains elements that provide meaning to the individual’s everyday life – hence defining meaning in life in terms of meaningfulness [1]. Sources of meaning can naturally vary over time and from person to person, therefore, subjective experiences, that is, focusing on what it is that causes us to experience meaningfulness [2], are important when studying these issues. Older adults themselves have highlighted social activities such as volunteering and meeting friends and family, in addition to different kinds of hobbies, as key carriers of meaning in everyday life [3, 4]. However, age-related changes in functional ability can constitute challenges and hinder a person from leading their life as desired [5]. Fortunately, today’s digitised society offers several online services that are regarded by the older users themselves as useful in carrying out meaningful activities [6].

The steady increase of internet users within the older population in Western countries (from 23% of the over 75-year-olds in 2010 to 69% in 2019 in Sweden) [7] has led to a growing number of studies from various disciplines exploring whether and how the use of internet-based services can be connected to and support older adults’ wellbeing [8]. Studies with this type of focus report that engaging in different online activities seems to have a generally advantageous impact on factors associated with mental health and wellbeing in later life, such as decreased loneliness [9], depression and anxiety [10], in addition to enhanced social connectedness and support [11]. However, besides its generally advantageous influence, the growing international evidence base of studies covering the connection between internet usage and loneliness [12] moreover indicates that different internet-based activities may yield varying influences (also negative) on the older individual’s wellbeing. Thus, it is of great importance to examine the content of older adults’ internet use in order to shed further light on this connection.

Based on previous research, both perceiving life as meaningful and using the internet seem to be beneficial for the psychological, emotional and social dimensions of wellbeing in later life [2, 11]. However, there is a limited amount of studies focusing on these two components and the association between them. Therefore, the aim of this article is to explore the association between internet use, the use of specific internet-based activities and perceiving life as meaningful, among older adults in two regions in Finland and Sweden. Further, the association analysis controls for potential covariates in terms of socio-demographic factors and self-rated health status.

## Methods

### Study design and data material

A population-based cross-sectional survey study was conducted in 2016 in Västerbotten (Sweden) and in Österbotten (Finland), as a part of the GErontological Regional DAtabase and Resource Centre (GERDA) project. The aim of the GERDA project was to gather information on older adults’ health and living conditions. The survey was posted to all individuals born in 1950, 1945, 1940, 1935 and 1930, living in rural areas and the city of Seinäjoki (Finland), to every second person living in the city of Vaasa (Finland) and to every third person within the same age cohorts living in the cities of Umeå (Sweden) and Skellefteå (Sweden) (in total 14,805 persons). The population register was obtained from the National Tax Board in Sweden and the Population Register Centre in Finland (for further details, see the GERDA Method Report [13] written for the study in 2010). In total, 9386 individuals participated in the survey study, giving a response rate of 63.40%.

### Measurements and variables

In Supplemental Tables I and II, the variables included in the study are presented in detail.

A set of socio-demographic variables were included in the analysis. These variables were recoded, and the cut-off points for the recoding process were chosen in accordance with other scientific articles based on the GERDA study [14]. The analysis also included a variable measuring self-rated health. The recoding process of the socio-demographic variables and self-rated health status are presented in Supplemental Table I.

Perceiving life as meaningful was measured with a single-item question that was dichotomised for the binary regression analysis. The dichotomising process, the survey question and response options are presented in Supplemental Table II.

Two different survey questions about internet use were included in the study. A variable measuring internet use, separating independent internet users from persons using internet with the support of others, and non-users, was created and dichotomised. Furthermore, five separate variables measuring internet activities, created from a multiple-answer question, were used to measure recent internet-based activities. These variables were also recoded into dummy variables and treated as separate categorical variables in the statistical analysis. The variables and the recoding process are given in Supplemental Table II.

### Ethics

The Regional Ethical Review Board in Umeå, Sweden, approved the data collection (05/084 & 2016/367–32). In Finland, ethical approval is not required for anonymous postal surveys. The ethical principles of the Helsinki Declaration were carefully followed during drafting of the article.

### Analysis

SPSS version 24 (IBM Corp, Armonk, NY, USA) was used for the statistical analyses. The distribution (%) of all included variables is reported by study regions in Supplemental Table III and the distributions (%) of perceiving life as meaningful among the socio-demographic variables, self-rated health and internet-related variables are presented in Table I. In order to explore the between-group comparison of perceived meaningfulness in relation to internet use and related activities and the socio-demographic variables and health status, a Pearson’s chi-square test was conducted (Table I). In order to further analyse the association between internet use, related internet-based activities and perceived meaningfulness in life, odds ratios (ORs) with 95% confidence intervals were calculated using a stepwise binary regression analysis (Table II). Furthermore, multiple independent variables (internet usage, internet activities, socio-demographic variables and self-rated health) were simultaneously analysed with the dependent variable (perceived meaningfulness), controlling for potential covariates among the independent variables [15]. Furthermore, the goodness of fit of the logistic regression models was tested using the Hosmer and Lemeshow test, which is commonly used for assessing whether there is agreement between the observed and the expected frequencies in model populations in logistic regression analysis [15].

**Table I.** The distribution of experiencing life as meaningful among all the included variables and Pearson’s chi-square test presenting between-group comparison of perceived meaningfulness.

	*Not* *meaningful* *(%)*	*Meaningful* *(%)*	*All* *(%)*	*χ* ^2^
Gender				
Men	847 (20.1)	3377 (79.9)	4948 (46.1)	*p* = .806
Women	982 (19.8)	3966 (80.2)	4948 (53.9)	
Age				
65	338 (12.4)	2379 (87.6)	2717 (29.7)	*p* ⩽ .001
70	457 (16.3)	2349 (83.7)	2806 (30.7)	
75	363 (21.9)	1291 (78.1)	1654 (18.1)	
80	362 (28.7)	899 (71.3)	1261 (13.8)	
85	308 (43.0)	408 (57.0)	716 (7.8)	
Study region				
Finland	977 (19.9)	3943 (80.1)	4920 (53.6)	*p* = .821
Sweden	853 (20.0)	3402 (80.0)	4255 (46.2)	
Marital status				
Single	783 (32.7)	1613 (67.3)	2396 (26.4)	*p* ⩽ .001
In a relationship	1031 (15.4)	5663 (84.6)	6694 (73.6)	
Educational level				
Low educational level	843 (25.1)	2515 (74.9)	3358 (37.1)	*p* ⩽ .001
Middle educational level	625 (18.7)	2843 (81.3)	3495 (38.6)	
High educational level	307 (13.9)	1894 (86.1)	2201 (24.3)	
Income level				
Low	598 (26.3)	1677 (73.7)	2275 (25.6)	*p* ⩽ .001
Middle	825 (20.0)	3310 (80.0)	4135 (46.5)	
High	340 (13.7)	2143 (86.3)	2483 (27.9)	
Self-rated health				
Moderate/poor	1162 (35.4)	2122 (64.6)	3284 (36.1)	*p* ⩽ .001
Good	434 (14.8)	2496 (85.2)	2930 (32.2)	
Very good	208 (7.2)	2681 (92.8)	2889 (31.7)	
Internet use				
Not internet users	866 (30.2)	2005 (69.8)	2871 (32.8)	*p* ⩽ .001
Internet users with support	107 (17.2)	516 (82.8)	623 (7.1)	
Internet users	839 (14.3)	5048 (85.6)	5887 (67.2)	
Internet-based activities				
Instrumental use	694 (14.1)	4226 (85.9)	4920 (53.6)	*p* ⩽ .001
Informational use	677 (14.0)	4167 (86.0)	4844 (52.8)	*p* ⩽ .001
Leisure/Entertainment	383 (12.7)	2639 (87.3)	3022 (32.9)	*p* ⩽ .001
Social network and support	499 (13.1)	3300 (86.9)	3799 (41.4)	*p* ⩽ .001
Other activities	42 (13.7)	264 (86.3)	306 (3.3)	*p* = .006

**Table II.** Odds ratio and their 95% confidence intervals of having meaning in life according to four logistic regression models as well as the result from the Hosmer and Lemeshow test of goodness of fit.

	All *N* = 8758			
	Model 1 Internet usage (three indicators)	Model 2 Internet usage + socio-demographic variables + self- rated health	Model 3 Internet-based activities	Model 4 Internet-based activities + Socio-demographic variables + self- rated health
Internet use				
Not internet users	1.00	1.00		
Internet users with support	2.083 (1.667–2.603)	1.418 (1.104–1.821)		
Independent internet users	2.674 (2.392–2.990)	1.273 (1.100–1.474)		
Internet-based activities				
Instrumental use			1.00	1.00
			1.417 (1.229–1.635)	1.009 (0.858–1.187)
Informational use			1.00	1.00
			1.353 (1.163–1.574)	1.004 (0.849–1.188)
Leisure/entertainment			1.00	1.00
			1.301 (1.121–1.511)	1.192 (1.015–1.401)
Social network and support			1.00	1.00
			1.307 (1.126–1.517)	1.73 (0.996–1.382)
Other activities			1.00	1.00
			1.294 (0.926–1.807)	1.436 (0.992–2.078)
Socio-demographic variables				
Gender				
Men		1.00		1.00
Women		1.559 (1.372–1.773)		1.494 (1.318–1.694)
Age				
65		1.00		1.00
70		0.862 (0.730–1.018)		0.875 (0.743–1.032)
75		0.757 (0.630–0.910)		0.779 (0.650–0.934)
80		0.677 (0.554–0.827)		0.689 (0.567–0.837)
85		0.481 (0.380–0.608)		0.468 (0.374–0.585)
Marital status				
Single		1.00		1.00
In a relationship		2.282 (2.002–2.601)		2.318 (2.042–2.632)
Study region				
Finland		1.00		1.00
Sweden		1.021 (0.905–1.152)		1.010 (0.898–1.137)
Educational level				
Low		1.00		1.00
Middle		0.961 (0.837–1.104)		0.989 (0.865–1.131)
High		1.081 (0.903–1.295)		1.055 (0.882–1.261)
Income level				
Low		1.00		1.00
Middle		1.319 (1.144–1.520)		1.276 (1.112–1.464)
High		1.409 (1.165–1.703)		1.383 (1.148–1.665)
Self-rated health				
Moderate/poor		1.00		1.00
Good		2.657 (2.316–3.046)		2.659 (2.326–3.039)
Very good		5.517 (4.640–6.559)		5.440 (4.594–6.442)
Hosmer and Lemeshow goodness-of-fit	Model 1 *χ*^2^ = 0.000, df = 1, *p* = 1.00	Model 2 *χ*^2^ = 10.028, df = 8, *p* = .263	Model 3 *χ*^2^ = 9.588, df = 6, *p* = .143	Model 4 *χ*^2^ = 6.598, df = 8, *p* = .581

The missing values in the collected data ranged from *N* = 3 (0.003%) to 437 (4.7%), where the lowest number was for gender and the highest for internet use. The missing values for perceiving life as meaningful were 211 (2.2%). A missing value analysis was performed for all the variables included in the analysis, which displayed a random pattern. The complete dataset can be accessed upon request.

## Results

### Descriptive statistics

In total, 46.6% of the participants were from Sweden and 53.4% from Finland (see Supplemental Table III). The sample had a higher representation of women and the vast majority of the study participants were in a relationship when the study was conducted. Moreover, 59.6% of the whole study sample were using the internet independently. The most used internet-based activity among the internet-using sample was usage related to practicalities (instrumental use), closely followed by usage related to information gathering (informational use). Some 63.8% (cases) of the internet users had been using internet for social networking and support during the last month, and 50.7% (cases) for leisure and entertainment. Only 5.2% (cases) indicated that they had recently used the internet for other activities. It is, however, important to keep in mind that the internet activities were measured with a multiple-answer question and that the older adults indicated several internet activities. Overall, 80.1% of the total sample perceived their life as meaningful.

### The association between internet use, internet-based activities and perceived meaningfulness in life

Statistically significant differences were found for all socio-demographic variables, except for gender and study regions, and health status with regard to perceiving life as meaningful (Table I). Significant differences between independent internet users, internet users with support, and non-users were detected from the data analysis: over 85% within the group of independent internet users and over 80% of the internet users with support perceived their life as meaningful, compared with 70% of the group of non-users.

The logistic regression analysis (Table II) further explored the association between internet use and related internet activities and perceived meaningfulness in later life in four steps. Four regression models are presented, the final model being the most advanced. The indicated ORs, obtained from the analysis, predicted the differences in perceiving life as meaningful among the different groups under study (e.g. independent internet users, internet users with support vs. non-users).

The first step of the analysis explored the association between internet use and perceived meaningfulness. The results indicated that there was a statistically significant association between using the internet both independently and with the support of others and perceiving life as meaningful (Model 1 in Table II). Both independent internet users and users that received support had more than two times higher odds of perceiving life as meaningful, compared with non-users.

In the next step (Model 2 in Table II), a set of socio-demographic variables together with the variable measuring self-rated health status were added to the analysis. The results indicated that using the internet both independently and with support had statistically significant associations with perceived meaningfulness – this was also the case after adjusting for the added variables. Perceived meaningfulness had several significant covariates among the added variables such as gender, marital status, income level and self-rated health (see Table II, Model 2 for more details). For instance, the older respondents who rated their health status as very good had five times higher odds of perceiving their life as meaningful, compared with respondents with poor self-rated health. Similar association patterns were found for income level.

The third step of the analysis explored the association between different internet-based activities and perceived meaningfulness. In Model 3 (Table II), the variable measuring general internet use in Models 1 and 2 was replaced by the five variables measuring internet-based activities (i.e. internet use content). The analysis showed that all of the internet-based activities under study had statistically significant associations to perceiving life as meaningful, compared with not engaging in them.

The same set of socio-demographic variables and self-rated health as in the analysis conducted in Model 2 were added to the final step of the analysis (Model 4 in Table II). After adjusting for these variables, only internet-based activity leisure/entertainment showed a statistically significant association with perceived meaningfulness. However, the OR was very close to being statistically significant for social network and support, as well as for the ‘other activities’ variable. Several significant covariates were found among the socio-demographic variables (gender, the same age groups as in Model 2, marital status, income level) and self-rated health status. Thus, these variables seems to partially explain the association found between using the internet for leisure/entertainment activities and perceiving life as meaningful.

The values of the Hosmer and Lemeshow goodness-of-fit statistics showed that the models used for the logistic regression analysis fit the data material (Table II).

## Discussion

This study adds to the public health field by highlighting evaluative aspects of older adults’ wellbeing (i.e. focusing on the subjective dimension of positive mental health as opposed to, for example, mental ill health) in relation to internet use and related everyday internet activities. Understanding the association between internet use and experienced wellbeing in later life is becoming increasingly important due to the increased presence of digital technology in everyday life and recent large public investments in welfare technology and digitalisation in the Nordic countries [16].

Between-group comparisons of older adults perceiving and not perceiving life as meaningful identified a significant difference with regard to internet use. Using the internet independently and with the support of another person significantly increased the odds of perceiving life as meaningful after adjusting for socio-demographic variables and self-rated health. The complex association between internet use and different components of wellbeing in later life has increasingly been explored in studies, which have indicated positive influences [11, 17], but also no direct effects [18]. In turn, the present evidence base suggests that internet use might have an indirect effect on older adults’ wellbeing, hence, the connection between internet use and wellbeing in later life might be moderated by other factors such as offline social interaction [19]. Thus, the significantly increased odds found in this study, of perceiving life as meaningful for the older adults who used the internet with the support of another person, might be related to the offline social interaction that occurs between persons when using the internet together. The analysis additionally demonstrated that the association between perceived meaningfulness in life and internet use have a number of statistically significant covariates in terms of socio-demographic variables and self-rated health. However, neither these variables nor the offline social interaction from receiving support using the internet can alone explain the association between internet use and perceived meaningfulness in life, since the independent internet use variable also demonstrated a statistically significant association after adjusting for socio-demographic variables and self-rated health status.

Based on previous study findings, the things you do on the internet (activities) seem to matter when it comes to the connection to wellbeing [20]. In an attempt to further explore and clarify this association, this study also tested specific internet-based activities’ associations with perceived meaningfulness in life. The results further demonstrated that, after controlling for socio-demographic variables and self-rated health status, leisure/entertainment activities were the only internet-based activities with a statistically significant association with perceiving life as meaningful. Previous studies investigating internet use for leisure/entertainment activities and an association with different aspects of wellbeing found similar results [21], where using the internet for leisure activities contributed to increased life satisfaction and decreased depression levels. In addition, qualitative findings [6] describe the use of the internet for leisure activities as rewarding and as something that expands the sphere of older adults’ everyday life. These functions were specifically highlighted because they were driven by the older persons’ own interests, unlike online banking or healthcare services that were experienced as being forced upon them by society.

How then are internet use and perceived meaningfulness in later life linked? The results of the study indicate that internet use and related internet-based activities (as well as various socio-demographic variables and health status) seem to matter when it comes to perceiving later life as meaningful. However, more studies, preferably longitudinal, are needed in order to explore causal interference between them. One possible link between internet use and perceived meaningfulness could be based on online services facilitating and increasing the opportunities for older adults to engage in activities perceived as meaningful. Specifically, the internet could be seen as a tool, facilitating engagement in meaningful activities; and participating in meaningful activities could, in turn, contribute to perceiving life as meaningful, as suggested by previous studies [22]. Studies exploring whether older persons with limitations in daily, social and leisure activities are enjoying the benefits of using internet-based activities compared with persons with no limitations are needed to further investigate this suggested link.

## Study limitations and strengths

One limitation with the study at hand was its cross-sectional design. The main weakness with this type of design is that determining causality is not possible. However, a benefit of logistic regression analysis is that it allows the researcher to study group differences of the variables while controlling for other potential covariates. By controlling for socio-demographic variables and health status, the study at hand recognises that several factors may be influencing the association between internet use and perceived meaningfulness in life. These factors were, however, not the main outcome of this specific study examining the link between internet use and wellbeing.

Logistic regression analysis demands artificial grouping of the study sample, which could be seen as an additional limitation with the method. The authors are aware of that the cut-off points used in the analysis are rough and that data as well as the nuances of some of the variables might have been lost during the recoding process. However, as the focus of this study was on categorical variables, not dividing the sample into groups (or using too many groups) could have led to a loss of statistical power in the analysis, as there would have been too small a number of participants in some of the cells [23]. Therefore, the somewhat artificial grouping of the sample in the present study can be seen as reasonable.

The survey questions regarding internet use and meaningfulness in life were not optimal. The number of missing values (437) (even though indicating a random missing pattern) for the internet variable could be seen as an indication of the internet questions being difficult to interpret. It should also be kept in mind, in the interpretations of the study findings, that the internet-based activities were measured by a multiple-answer question, and that the respondents frequently indicated several internet-based activities. Moreover, perceived meaningfulness in life in this study was measured with a single-item question. Undoubtedly, a single-item question cannot capture all the components of perceived meaning in life. However, this question may have been experienced by the respondents as quite easy to grasp in an otherwise rather extensive, all-embracing postal survey (82 questions) covering several scales and measurements. In addition, in a recent review identifying and appraising existing instruments to evaluate mental wellbeing in old age [24], no existing instruments regarding evaluative and experienced wellbeing (e.g. experienced meaning) were recommended due to being poor quality or having limited usability owing to the lack of language translations. Therefore, the use of a single-item question can be justified in this study. Consequently, there is a need to develop instruments for measuring both internet-based activity as well as evaluative and experienced wellbeing in later life.

Finally, the extensive postal survey might also have created a risk of bias due to self-selection sampling. Older adults with more severe health problems may not have completed the survey (due to the high number of questions, for example), which might have resulted in both higher numbers of persons perceiving their life as meaningful, and internet users within the study sample than if more persons with a poorer health status had participated in the survey.

Overall, the limitations of the study could be argued to be compensated for by its strengths of being based on larger, high-quality survey data, combining two Nordic geographical regions, and covering various groups within the heterogeneous group of older adults. In addition, the study at hand adds to the limited body of research examining the often-overlooked evaluative and experienced dimensions of wellbeing in an ageing population.

## Supplemental Material

sj-pdf-1-sjp-10.1177_1403494820987459 – Supplemental material for Nordic population-based study on internet use and perceived meaningfulness in later life: How they are linked and why it matters Click here for additional data file. Supplemental material, sj-pdf-1-sjp-10.1177_1403494820987459 for Nordic population-based study on internet use and perceived meaningfulness in later life: How they are linked and why it matters by Emilia W.E. Viklund, Ingeborg Nilsson and Anna K. Forsman in Scandinavian Journal of Public Health
