# Supplementary material for: Nordic population-based study on internet use and perceived meaningfulness in later life: How they are linked and why it matters
Source: Scand J Public Health. 2021 Feb 15;50(3):381–8. doi: 10.1177/1403494820987459 (PMC9096589; doi:10.1177/1403494820987459)
Supplement: sj-pdf-1-sjp-10.1177_1403494820987459 – Supplemental material for Nordic population-based study on internet use and perceived meaningfulness in later life: How they are linked and why it matters [file sj-pdf-1-sjp-10.1177_1403494820987459.pdf]

Supplemental Table I. Socio-demographic variables, self-rated health status, and the recoding process.

Variables	Response options	Recoded variables used in analysis
Socio-demographic variables		
Gender	Men	*
	Women	
Age	65	*
	70	
	75	
	80	
	85	
Study region	Finland	*
	Sweden	
Marital status	Unmarried	Single
	Divorced	
	Widow/Widower	
	In a relationship	In a relationship
	Living with a partner	
	Married	
Educational level	6 years or less (elementary school)	Low
	7 years or more (high school, upper secondary school, vocational training)	Middle
	University or university of applied science education	High
Income level	SEK 0–5000 /€ 0–500	Low
	SEK 5001–10,000 /€ 501–1000	
'Your monthly income after taxes?'	SEK 10,001–15,000 /€ 1001–1500	Middle
	SEK 15,001–20,000 /€ 1501–2000	
	More than SEK 20,000 /€ 2000	High
Self-rated health		
'In general, how would you describe your health?'	Bad	Fair/bad
	Fair	
	Good	Good
	Very good	Very good
	Excellent	

*The original variables were used in the analysis.

Supplemental Table II. Meaningfulness in life and internet variables and the recoding process.

Variables	Response options	Recoded variables used in the analysis
Meaningfulness in life		
'How meaningful do you perceive your life at the moment?'	Very meaningless	Not meaningful
	Relatively meaningless	
	Hard to tell	
	Relatively meaningful	Meaningful
	Very meaningful	
Internet use		
'Are you using the internet (via computer, tablet computer, smart phone or similar device)?'	No, someone else is managing my errands on the internet for me	Not internet users
	No, I do not use the internet	
	Yes, with support from others	Internet user with support
	Yes, independently	Internet users
Internet-based activities		
'Have you been using the internet for the following purposes during the last month?'	Utilities[1]	Instrumental use
	Work or studies	
	News updates or information gathering[2]	Informational use
	Leisure/entertainment[3]	Leisure/entertainment
Multiple answers were allowed	Communication with relatives and/or friends[4]	Social networking and support
	Communication to create new contact[5]	
	Support groups	
	What else?[6]	Other activities

[1]Bank, travel arrangements, social security.
[2]Newspapers, news forums.
[3]Music, movies, games, forums connected to interests.
[4]Email, Skype, Facebook, social networks.
[5]Facebook, forums, online dating.
[6]Blogs, sports, games, church services.

Supplemental Table III. Distribution (%) of the socio-demographic variables, self-rated health, perceived meaningfulness and internet use among older adults in Finland and Sweden.

	Sweden<br>*N* = 4375<br>(46.6)	Finland<br>*N* = 5011<br>(53.4)	All<br>*N* = 9386	
Gender				
Men	2123 (48.6)	2211 (44.1)	4334 (46.2)	
Women	2249 (51.4)	2800 (55.9)	5049 (53.8)	
Age				
65	1207 (27.6)	1544 (30.9)	2751 (29.4)	
70	1345 (30.8)	1518 (30.4)	2863 (30.6)	
75	864 (19.8)	828 (16.6)	1692 (18.1)	
80	599 (13.7)	701 (14.0)	1300 (13.9)	
85	356 (8.1)	403 (8.1)	759 (8.1)	
Marital status				
Single	1186 (27.5)	1285 (25.8)	2471 (26.6)	
In a relationship	3133 (72.5)	3695 (74.2)	6828 (73.4)	
Educational level				
Low	1980 (45.7)	1512 (30.4)	3472(37.5)	
Middle	1389 (32.4)	2171 (43.7)	3560 (38.5)	
High	942 (22.0)	1283 (25.8)	2225 (24.0)	
Income level				
Low	1201 (28.3)	1129 (23.5)	2330 (25.7)	
Middle	2045 (48.2)	2161 (44.9)	4206 (46.5)	
High	997 (23.5)	1519 (31.6)	2516 (27.8)	
Self-rated health				
Moderate/poor	1471 (34.0)	1905 (38.4)	3376 (36.3)	
Good	1468 (33.9)	1512 (39.5)	2980 (32.1)	
Very good	1387 (32.1)	1547 (31.2)	2934 (31.6)	
Perceiving life as meaningful				
Not meaningful	835 (20.0)	977 (19.9)	1830 (19.9)	
Meaningful	3402 (80.0)	3943 (80.1)	7345 (80.1)	
Internet use				
Not internet users	1325 (31.4)	1651 (34.9)	2976 (33.3)	
Internet users with support	341 (8.1)	296 (6.3)	637 (7.1)	
Internet users	2550 (60.5)	2786 (58.9)	5336 (59.6)	
Internet-based activities (multiple-answer question)			*N* and % responses	% cases
Instrumental use	2388 (54.6)	2598 (51.8)	4986 (53.1)	(82.8)
Informational use	2365 (54.1)	2540 (50.7)	4905 (52.3)	(81.4)
Leisure/entertainment	1492 (34.1)	1562 (31.2)	3054 (32.5)	(50.7)
Social network and support	1879 (42.9)	1967 (39.3)	3846 (41.0)	(63.8)
Other activities	107 (2.4)	207 (4.1)	311 (3.3)	(5.2)
